# Progress of the Cholera

**Published:** 1848-11

**Authors:** 


*Progress of the Cholera.*—The following inteliigence with respect to the appearance of the Cholera in England, and its progress in other parts of Europe, is taken from newspapers brought by the steamer Europa, which arrived at New York on the 25th ult.

Liverpool, Oct. 14.—*The Cholera*—Several cases of this dreadful disease are reported to have occurred in London during the past week. The Times of Tuesday thus sums up the cases; 5 in Chelsea; 3 at Rotherhithe; 2 in the city of London; 2 in Spitalfields; 2 in Whitechapel; 2 in Bermondsey: 2 in Horsley-down; inclusive of those at Woolwich, 26 fatal cases have been reported.

At Sunderland two deaths have occurred on board a vessel arrived from Hamburgh. One of the deceased men was a great drunkard.

At Edinburgh, on Sunday, there had been 14 ascertained cases, of which seven terminated fatally. Seven cases had also occurred at Newhaven.

The disease made its appearance almost cotemporaneously in Sunderland and in the low lying districts below London Bridge. In both places the first cases were those of intemperate sailors who came from Hamburgh and were attacked by the malady on the voyage. As regards Edinburgh, the origin of the disease is left in doubt. The official report of the Registrar-General in London reported 13 cases up to Saturday last. In Edinburgh, up to the latest report, there had been 25 cases, 20 of which had proved fatal. Up to Wednesday in the present week the number of cases in London is alleged to be about 20, but a daily official report is not yet issued.

The 13 cases in London above mentioned are thus particularized in the official returns. They all occurred during the week ending the 7th inst. From that date to the 14th, the disease seems not to have made much progress:

"In Old street (sub-district,) St. Luke, at 39, Rahere street, wife of a gentleman, 59 years, disease of the bowels simulating Asiatic cholera (38 hours' duration.) In south sub-district, west London, F., 27 years, cholera (13 hours' duration.) In St. Bartholomew's Hospital, west London, M., about 40 years, Asiatic cholera. In Town (sub-district) Bethnal-green, at No. 4, Cheshire street, a weaver F., 21 years, enlargement of the heart (12 months' duration,) cholera-spasmodic (12 hours.) In Spitalfields, Whitechapel, M., 23 years, cholera (12 hours' duration.) In Whitechapel north, a girl, 4 years, English sporadic cholera (seven days' duration.) In St Paul (sub-district,) St. George-in-the-East, M., 38 years, cholera (2 days duration.) In Mile-end, Old town, Lower Stephney, M., 47 years, cholera (36 hours duration.) Mr. Castleden's (the registrar) note: Mr. Todd, the surgeon in this case, certifies that the above was a case of Asiatic cholera; and the informant states that her father (the deceased) got up on Thursday morning, about four o'clock, with a bowel complaint, for which he took some gin and ginger, and then went to his work on board ship, but which he was compelled to relinquish about mid-day. He returned home in a cab, and died yesterday morning at four o'clock. The medical gentleman was in close attendance to the very last. In Lambeth Church, 2nd part, (sub-district) at Orsett street, daughter of a chair-maker, aged 11 months, cholera, (3 days duration): convulsions (1 hour.) Mr. W. H. Wheatley, the registrar, states, "that at one end of the street mentioned above, in the centre of the road, is an open drain, which is very offensive at times—drainage to houses very bad. Scarlatina has been very prevalent there." In Rotherhithe, a boy, 11 years old, cholera (19 hours duration.) In same sub-district, F., 38 years, cholera (19 hours duration.) In same sub-district, a girl, 2 years, cholera (2 days duration.) In Greenwhich West (sub-district,) F., 37 years, cholera (4 days duration.)

London, Oct. 13.—The highly favorable change in the weather, it is to be hoped, has checked the progress of fatal cholera cases in the metropolis. Only one fatal case was reported yesterday—that of a person in the Tower. The attacks of diarrhoea were reported to be numerous and severe, with, however, very satisfactory statements of the success of the treatment recommended for checking the disease at once in the first stage, or in its premonitory symptoms.

Hamburgh.—Authentic accounts state that the disease is still on the increase at Hamburg. From the 1st September, when it broke out, to the 26th, there were 1330 cases, of which 650 died, 302 recovered, and 387 were under treatment. There is a great deal of sickness on board the English ships lying at Hamburg.

Berlin.—Up to the 30th ult, there have been 1704 cases of cholera in Berlin, 1102 of which terminated fatally, 302 reported as cured, and 390 still doubtful.

St. Petersburg.—The last accounts from St. Petersburg give about 19 new cases, and seven or eight deaths daily.

Aleppo and Damascus.—The ravages of the pestilence at Aleppo and Damascus are stated to have been frightful, especially at Damascus, where not fewer than 10,000 persons are supposed to have died within twenty days preceding the 26th of August.

